# Two Cases of NUT Carcinoma of the Lung: Same Name With Different Appearance: A Case Report

**DOI:** 10.1155/crip/9648746

**Published:** 2026-10-06

**Authors:** Eriko Atsumi, Hidenori Kawasaki, Isoko Owan, Futoshi Higa, Hajime Aoyama, Hirofumi Matsumoto

**Affiliations:** ^1^ Division of Pathology NHO Okinawa Hospital Ginowan Okinawa Japan; ^2^ Division of Surgery NHO Okinawa Hospital Ginowan Okinawa Japan; ^3^ Division of Pulmonary Medicine NHO Okinawa Hospital Ginowan Okinawa Japan; ^4^ Department of Pathology Heart-Life Hospital Nakagami-gun Okinawa Japan; ^5^ Department of Pathology Nakagami Hospital Okinawa City Okinawa Japan

**Keywords:** case report, immunohistochemistry, NUT carcinoma, small round cell tumor, squamous epithelium

## Abstract

NUT carcinoma is a poorly differentiated tumor defined by nuclear protein in testis (*NUTM1*) gene rearrangement. Histologically, it is characterized by a primitive small round cell proliferation with abrupt squamous differentiation, although the extent of squamous differentiation varies among cases. Inconspicuous squamous epithelium may result in misdiagnosis as lymphoma or other small round cell tumors. In this study, we present two cases of NUT carcinoma: (1) a man in his 20s with a right hilar and middle lobe mass, in whom a lymph node biopsy was performed followed by autopsy; and (2) a woman in her 30s with a left lung mass that was surgically resected. Both cases showed primitive small round cell proliferation; however, they differed in both morphological and immunohistochemical features. Morphologically, the appearance and extent of squamous differentiation varied between the two cases. The first case exhibited abrupt squamous epithelium intermingled with the round cell component, whereas the second exhibited tiny foci of squamous epithelium, with the remainder composed of primitive round cells. Regarding immunohistochemistry, the first case showed diffuse keratin positivity, whereas the second showed almost no staining. Based on these results, the first case was considered typical NUT carcinoma, whereas the second was difficult to diagnose due to limited squamous epithelium. Although the key diagnostic feature of NUT carcinoma is reported to be abrupt squamous differentiation, many cases do not exhibit this feature, which may represent a diagnostic pitfall. There are also pitfalls in immunohistochemistry: keratin, which confirms epithelial origin, may be negative; p40, which highlights squamous differentiation, may also be negative; and TTF‐1 and neuroendocrine markers may be positive. This report compares these two cases morphologically and immunohistochemically and highlights the diagnostic challenges of NUT carcinoma with scant squamous components. Additionally, related literature is reviewed, and key diagnostic points, including immunohistochemistry, are discussed.

## 1. Introduction

NUT carcinoma is a poorly differentiated, highly aggressive tumor defined by nuclear protein in testis (*NUTM1*) gene rearrangement, showing partial histological differentiation toward squamous cell carcinoma [1, 2]. Although characteristic small round cells with abrupt keratinization aid in diagnosis, squamous differentiation is reported in only about 33% of cases [3]. Therefore, most cases lack abrupt keratinization, making diagnosis more challenging than in typical cases. In this study, we report two cases of NUT carcinoma: one with typical abrupt keratinization and one with minimal squamous component, which is difficult to diagnose.

We present the following case report in accordance with the CARE checklist (File S1).

## 2. Case Presentation

### 2.1. Case 1

A man in his 20s with a history of bronchial asthma and no history of smoking presented with a dry cough and wheezing. Chest x‐ray revealed a right hilar tumor, and he was admitted to our hospital. Chest computed tomography (CT) revealed a tumor (3 cm) in the right middle lobe and a mass (6 × 7 cm) in the right hilar area (Figure 1). The right cervical, supraclavicular, and mediastinal lymph nodes were enlarged. A cervical lymph node biopsy was performed following a nondiagnostic transbronchial lung biopsy (TBLB).

**Figure 1:**
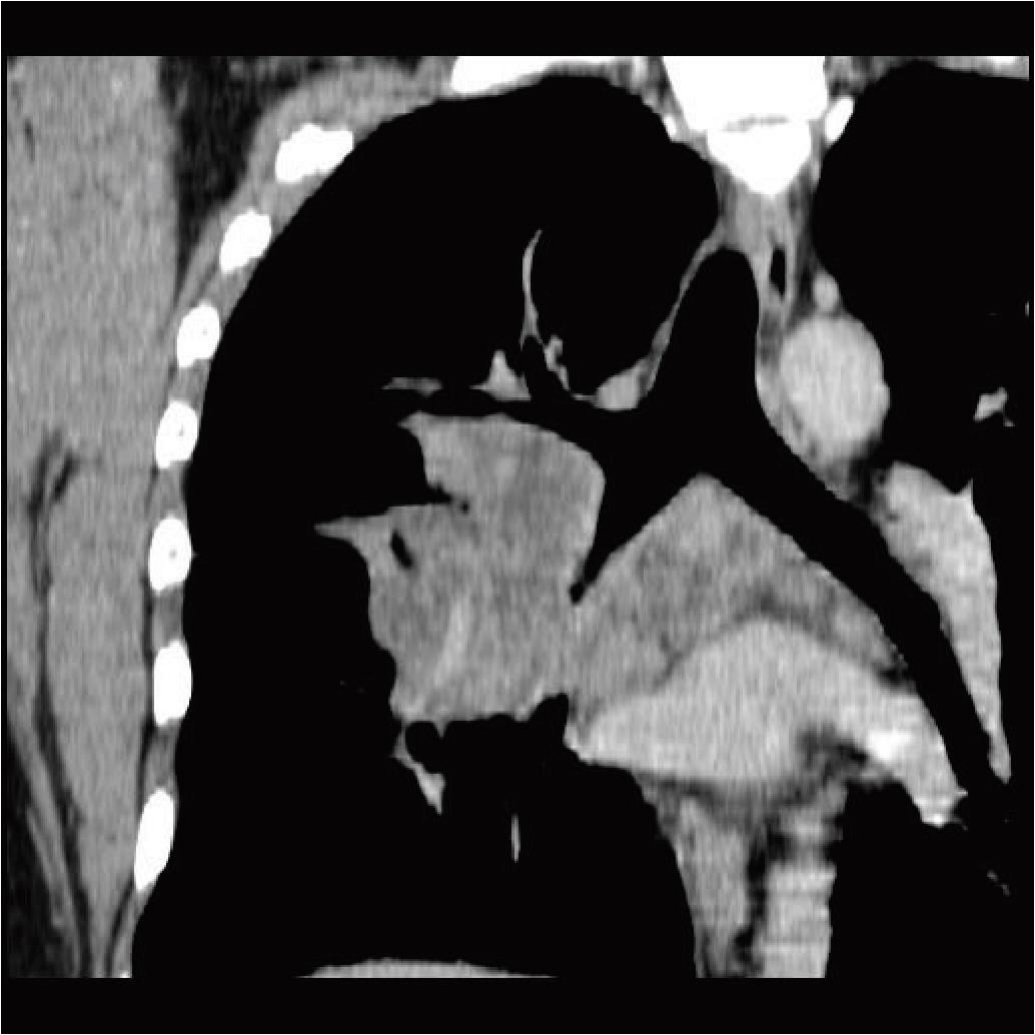
Chest computed tomography on admission demonstrates a right hilar tumor.

#### 2.1.1. Pathological Findings

The tumor exhibited a biphasic appearance, with one component composed of small round cells showing a high nucleus‐to‐cytoplasm (N/C) ratio and 1–2 small nucleoli. The other component consisted of squamous epithelium foci, in which the squamoid cells had abundant clear cytoplasm with minimal nuclear atypia. Squamous elements were intermingled with the small round cell component in an insular pattern (Figure 2). Immunohistochemistry of the small round cell component showed positivity for anti–pan‐cytokeratin antibody AE1/AE3 (Figure 3A), p63 (Figure 3B), p40, epithelial membrane antigen (EMA), carcinoembryonic antigen, and anticytokeratin antibody CAM5.2, but negativity for thyroid transcription factor‐1 (TTF‐1), neuroendocrine markers, myoepithelial markers, gross cystic disease fluid protein‐15, and cluster of differentiation (CD)15. The Ki‐67 labeling index was approximately 30% in the hotspot. Salivary gland tumors and unusual squamous cell carcinoma were considered in the differential diagnosis; however, the tumor was initially categorized as an unclassified carcinoma.

**Figure 2:**
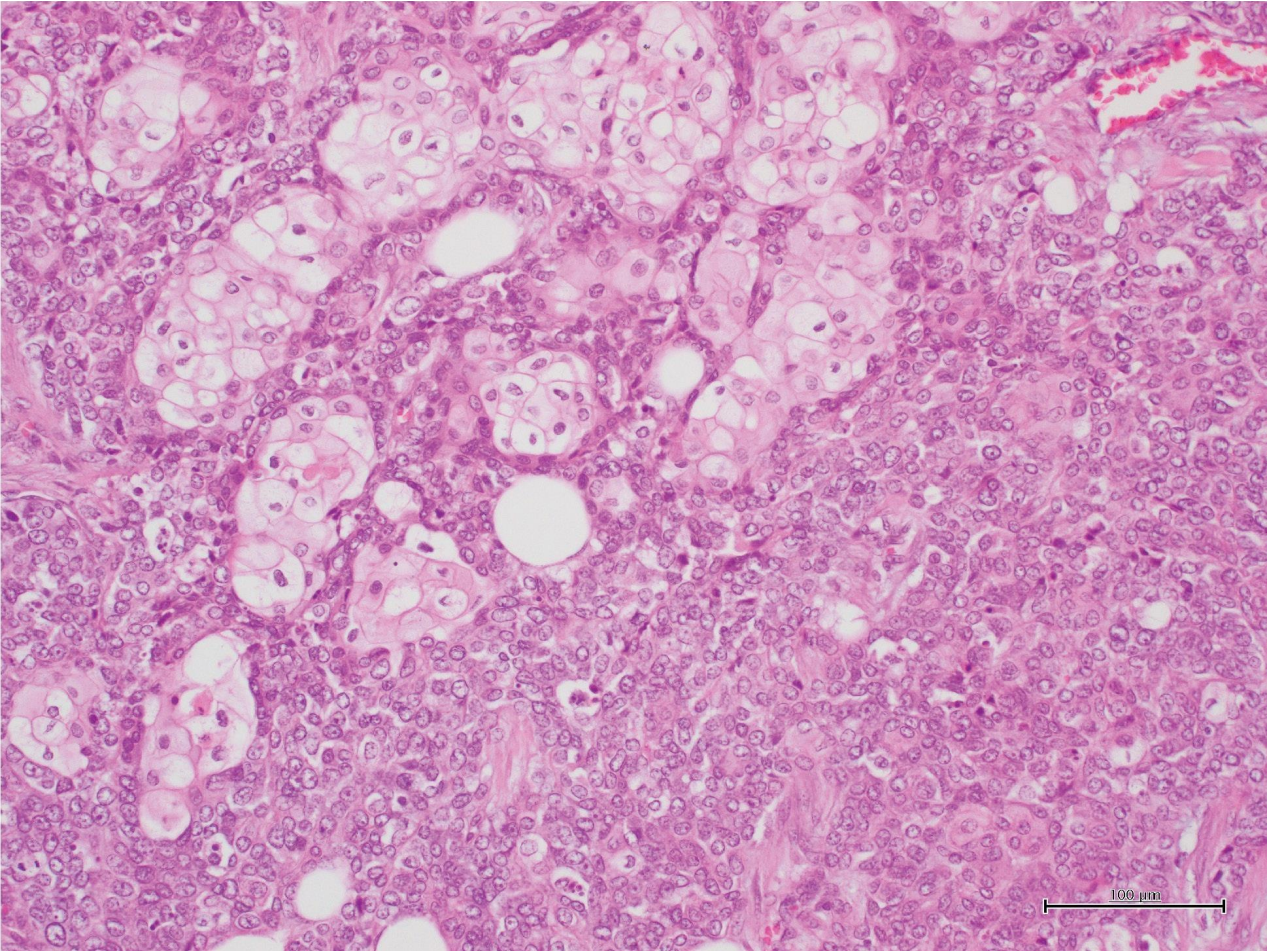
Microscopically, small round cells are admixed with foci of squamous differentiation (hematoxylin and eosin [H&E] stain).

**Figure 3:**
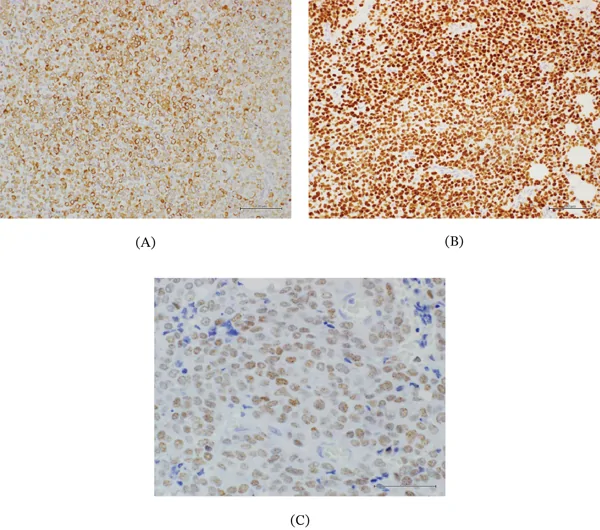
On immunohistochemistry, the small round cells are positive for (A) AE1/AE3 and (B) p63. Immunohistochemistry for NUT performed on the lymph node biopsy after 10 years of FFPE block storage was positive (C).

#### 2.1.2. Clinical Course

As the tumor was resistant to cisplatin and gemcitabine chemotherapy, the patient died 5 months after admission. Autopsy revealed a primary tumor in the right middle and lower lobes, with multiple metastases to the lymph nodes, liver, right kidney, right adrenal gland, and bones. Histologically, these tumors resembled the lymph node biopsy, although small round cells predominated. The autopsy diagnosis was undifferentiated lung carcinoma. However, after 10 years, regarding the patient′s prominent mediastinal lesions, the possibility of NUT carcinoma was raised as a differential diagnosis by the clinician. Immunohistochemistry for NUT (clone: C52B1; Cell Signaling Technology, Danvers, Massachusetts, United States) performed on the stored formalin‐fixed, paraffin‐embedded (FFPE) lymph node biopsy sample was positive (Figure 3C), resulting in a final diagnosis of NUT carcinoma.

### 2.2. Case 2

A woman in her 30s presented with back pain and cough. Chest CT revealed a tumor (10 cm) in the left hilar to lower lobe (Figure 4A). CT‐guided needle biopsy indicated a small round cell tumor. Malignant lymphoma was considered in the differential diagnosis; however, negative immunohistochemistry performed on the CT‐guided needle biopsy for leukocyte common antigen, CD3, and CD79a excluded this possibility. AE1/AE3, CAM5.2, TTF‐1, neuron‐specific enolase, CD56, and CD99 performed on the same specimen were also negative. As undifferentiated sarcomas remained in the differential diagnosis, a left pneumonectomy with pericardial resection was performed.

**Figure 4:**
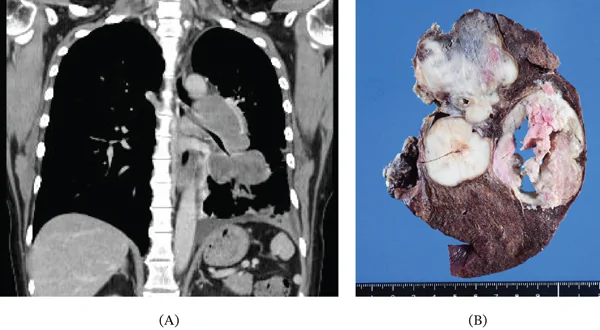
(A) Chest computed tomography on admission demonstrates a tumor located in the left hilar to lower lobe. (B) Macroscopically, the resected tumor is whitish and measured 10 × 7.5 × 6 cm.

#### 2.2.1. Pathological Findings

Macroscopically, the resected tumor was whitish and measured 10.0 × 7.5 × 6.0 cm (Figure 4B). Microscopically, discohesive small round cells proliferated diffusely with extensive necrosis (Figure 5A). These cells exhibited a high N/C ratio, with nearly naked nuclei, 1–2 nucleoli, and irregular nuclei contours. Small foci of polygonal cohesive cells proliferated in a sheet‐like pattern. The abundant cytoplasm and intercellular bridges of these polygonal cells indicated squamous differentiation (Figure 5B). Immunohistochemistry of the resected specimen showed that the discohesive tumor cells were positive for EMA, p63 (Figure 6A), and p40, but largely negative for AE1/AE3 (with only a few positive cells; Figure 6B) and completely negative for lymphoma and neuroendocrine markers. The Ki‐67 labeling index was approximately 50% in the hotspot. Additional sarcoma markers, including SMA, desmin, S‐100, MPO, CD68, and CD163, were negative. The extensive mediastinal involvement and focal keratin positivity raised the differential diagnosis of NUT carcinoma. Immunohistochemistry for NUT (clone C52B1; Cell Signaling Technology) was positive (Figure 6C), confirming the diagnosis of NUT carcinoma.

**Figure 5:**
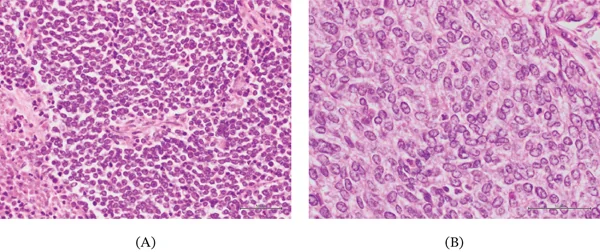
(A) Microscopically, small round cells proliferate diffusely in most areas with necrosis (H&E stain). (B) The squamous component shows polygonal cells with clear to eosinophilic cytoplasm (H&E stain).

**Figure 6:**
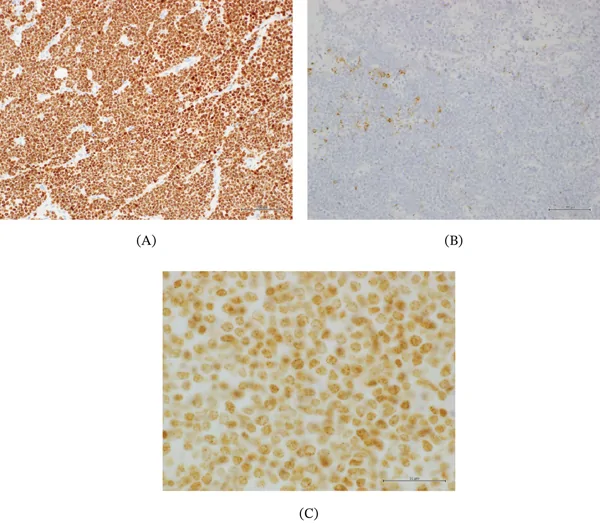
On immunohistochemistry, the small round cells are positive for (A) p63, but largely negative for (B) AE1/AE3. Immunohistochemistry for NUT is positive, showing a characteristic nuclear speckled pattern (C).

#### 2.2.2. Clinical Course

Approximately 5 months postoperatively, the patient developed massive left malignant pleural effusion, and positron emission tomography/CT revealed multiple metastases to the pleural cavity, lymph nodes, bones, and right ovary. She underwent two courses of chemotherapy with carboplatin, pemetrexed, and bevacizumab, which had a limited effect. Subsequently, she declined further chemotherapy and received palliative radiotherapy and metastatic ovarian tumor resection to prevent intestinal obstruction. The patient died 1 year later, and no autopsy was performed.

The comparison of the two cases is shown in Table 1.

**Table 1:** Comparison of the clinicopathological and immunohistochemical findings in the two cases of NUT carcinoma.

Finding	Case 1	Case 2
Clinical characteristics		
Age	20s	30s
Sex	Male	Female
Location	RML	LL
Treatment	Chemotherapy (cisplatin + gemcitabine)	Pneumonectomy, chemotherapy (carboplatin + pemetrexed + bevacizumab)
Pathological findings		
Morphology		
Squamous epithelium	Abundant	Little
Relation between squamous epithelium and small round cell component	Admixed	Adjacent
Immunohistochemistry		
AE1/AE3	+	Focal+
CAM5.2	+	—
EMA	+	+
p40	+	+
p63	+	+
NUT	+	+
HMW keratin (34*β*E12)	+	N/A
CK5/6	N/A	Only in squamoid area
CEA	Only in squamoid area	N/A
TTF‐1	—	—
CD34	N/A	—
CD56	—	—
Synaptophysin	—	—
Ki‐67 labeling index	30%	50%

Abbreviations: AE1/AE3, anti–pan‐cytokeratin antibody; CAM5.2, anticytokeratin antibody; CEA, carcinoembryonic antigen; CK, cytokeratin; EMA, epithelial membrane antigen; HMW, high molecular weight; LL, left lung; N/A, not assessed; NUT, nuclear protein in testis; RML, right middle lobe; TTF‐1, thyroid transcription factor‐1.

## 3. Discussion

NUT carcinoma, a poorly differentiated tumor defined by *NUTM1* rearrangement [1, 2], was first described in 1991 as thymic carcinoma or intrathoracic carcinoma with t(15;19) translocation, reported independently in Japan [4] and Australia [5]. In early 2000s, French et al. [6] identified the bromodomain‐containing protein 4 (*BRD4*)–*NUT* fusion gene, which results from the fusion of *NUTM1* with *BRD4*. Additional fusion gene partners, including *BRD3* [6], nuclear receptor binding SET domain protein 3 (*NSD3*) [7], zinc finger protein 532 (*ZNF532*) [8], and *ZNF592* [9], have also been identified. Among *NUTM1* rearrangements, *BRD4*–*NUT* is predominant, occurring in approximately 72%–78% of cases, followed by *BRD3*–*NUT* and *NSD3*–*NUT* [1, 3, 10, 11]. In addition to *BRD4*, several fusion partners encode *BRD4*‐interacting proteins that link *NUT* to *BRD4*, effectively generating the same *BRD4*–*NUT* oncogenic driver [1]. The *BRD4*–*NUT* fusion oncoprotein blocks differentiation by upregulating *MYC*, maintaining cells in an undifferentiated, proliferative state; this mechanism is considered the primary driver of carcinogenesis in NUT carcinoma [1, 12].

Clinically, NUT carcinoma was initially reported in young adults but is now recognized to affect all age groups, with no sex predilection [1]. It occurs most frequently in thoracic and mediastinal sites (50%–60%), followed by the head and neck (35%–41%), bones and soft tissues (6%), and other sites, including the salivary glands, pancreas, bladder, kidneys, and adrenal glands [3, 13]. Despite its highly aggressive nature, median survival varies by primary anatomical site and NUT fusion partner, with pulmonary and thoracic cases having the worst prognosis [3, 10]. Chau et al. [3] reported that thoracic NUT carcinoma has a significantly shorter median overall survival (OS) (4.4 months, regardless of NUT fusion partner) than nonthoracic NUT carcinoma (36.5 months for *BRD3*– or *NSD3*–*NUT*; 10 months for *BRD4*–*NUT*). Kloker et al. [10] reported that among 30 adult patients with NUT carcinoma, thoracic cases had a significantly shorter median OS than nonthoracic cases (182 days vs. 415 days).

Histologically, NUT carcinoma primarily consists of small‐ to intermediate‐sized monomorphous undifferentiated cells with a high N/C ratio. These cells proliferate diffusely or in nests and exhibit a discohesive pattern. Unlike small cell carcinoma, they do not show nuclear molding [1]. One morphological feature useful for diagnosis is abrupt keratinization or squamous differentiation, although this is present in only a minority of cases. Chau et al. [3] reported that among 124 patients, focal squamous pearls, stratification, or cell enlargement indicating squamous differentiation were present in only 33% of cases. Cho et al. [14] reported no keratinization in 10 cases, even in those with prominent squamoid features.

Immunohistochemistry is essential for distinguishing NUT carcinoma from histological mimics; however, some caution is warranted. First, NUT carcinoma frequently shows positivity for CD34 and may occasionally express synaptophysin or TTF‐1 [1]. Farooq et al. [15] reported that among thoracic NUT carcinoma cases, synaptophysin and CD56 were positive in 17% and 25% of patients, respectively, whereas TTF‐1 was positive in 31%, indicating that positivity for these markers does not exclude NUT carcinoma. Second, not all NUT carcinomas express p40 [15], despite evidence of squamous differentiation. In thoracic cases reported by Farooq et al. [15], p40 was positive in 63%, whereas p63 was positive in 85%. Therefore, when NUT carcinoma is suspected based on morphology, the inclusion of p63 is important if p40 is negative. Third, keratin staining represents another potential pitfall: although NUT carcinoma is epithelial, not all cases show keratin positivity. Farooq et al. [15] reported positivity rates of 81%, 82%, and 81% for AE1/AE3, CAM5.2, and any keratin (including AE1/AE3, CAM5.2, OSCAR, CK7, and CK20), respectively; therefore, approximately 20% of cases were keratin‐negative [15]. Keratin negativity can lead to misclassification as sarcoma. In our second case, AE1/AE3 was almost negative and CAM5.2 was negative, whereas the first case showed diffuse positivity for both (Table 1). Thus, NUT carcinoma should not be excluded based solely on negative keratin staining. Diagnosis relies on positive NUT protein immunohistochemistry (> 50% of tumor cells using clone C52B1) or molecular testing, including fluorescence in situ hybridization, reverse transcriptase polymerase chain reaction, or next‐generation sequencing [1]. NUT protein staining with C52B1 is 87% sensitive and highly specific, with only weak, focal staining observed in seminoma, dysgerminoma, and embryonal carcinoma [1]. In our first case, NUT staining was slightly weak, possibly related to the 10‐year storage of the FFPE block.

The differential diagnosis includes lymphoma, leukemia, small round cell tumors (including Ewing sarcoma or related tumors), small cell carcinoma, and SMARCA4‐deficient undifferentiated tumor [1]. When a cohesive growth pattern is present, basaloid squamous cell carcinoma and combined small cell carcinoma should also be considered in the differential diagnosis [1, 2].

Overall, the first case was considered typical, with small undifferentiated cells admixed with abrupt foci of squamous differentiation, whereas the second case was more challenging, showing only small foci of squamous differentiation at the periphery of large undifferentiated areas. No squamous differentiation was intermingled within the small round cell component. Although these tumors share the same name, their microscopic appearance differed according to the extent of the squamous component.

To date, no therapy has proven effective for the treatment of NUT carcinoma; however, clinical studies are ongoing, including those investigating inhibitors of the bromodomain and extraterminal domain [16–19], p300 [18], histone deacetylase [18, 19], and CDK9 [18, 19], which may enable effective treatment in future cases. Thus, it is important to recognize NUT carcinoma though clues are limited.

## Author Contributions

E.A. wrote and revised the manuscript. H.K., I.O., and F.H. managed the patients and acquired the clinical data. H.A. contributed to the diagnostic process. H.M. contributed to the diagnostic process and revised the manuscript.

## Funding

No funding was received for this manuscript.

## Disclosure

All authors have read and approved the final version of the manuscript before submission.

## Ethics Statement

Our institutional ethics committee approved this study (Approval No. 2024‐24). All medical interventions were conducted in accordance with the Declaration of Helsinki.

## Consent

Written informed consent for publication was obtained from the next of kin of both patients.

## Conflicts of Interest

The authors declare no conflicts of interest.

## Supporting information


**Supporting Information** Additional supporting information can be found online in the Supporting Information section. File S1: CARE checklist. The completed CARE checklist of this case report is provided as a supporting file.

## Data Availability

The data that support the findings of this study are available from the corresponding author upon reasonable request.

## References

[1] French C. A. , Badve S. , den Bakker M. A. , and Jain D. , NUT Carcinoma of the Thorax, WHO Classification of Tumours Editorial Board, WHO Classification of Tumors, Thoracic Tumours, 2021, 5th edition, WHO.

[2] Travis W. D. , Nicholson A. G. , Geisinger K. R. , and Brambilla E. , Travis W. D. , Nicholson A. G. , Geisinger K. R. , and Brambilla E. , Unclassifiable and Miscellaneous Carcinomas, AFIP Atlas of Tumor Pathology Series 4, Tumors of the Lower Respiratory Tract, 2019, American Registry of Pathology, 10.55418/9781933477466-13.

[3] Chau N. G. , Ma C. , Danga K. , Al-Sayegh H. , Nardi V. , Barrette R. , Lathan C. S. , DuBois S. G. , Haddad R. I. , Shapiro G. I. , and Sallan S. E. , An Anatomical Site and Genetic-Based Prognostic Model for Patients With Nuclear Protein in Testis (NUT) Midline Carcinoma: Analysis of 124 Patients, JNCI Cancer Spectrum. (2019) 4, no. 2, pkz094, 10.1093/jncics/pkz094, 32328562. PMC7165803 32328562

[4] Kubonishi I. , Takehara N. , Iwata J. , Sonobe H. , Ohtsuki Y. , Abe T. , and Miyoshi I. , Novel t (15; 19)(q15; p13) Chromosome Abnormality in a Thymic Carcinoma, Cancer Research. (1991) 51, no. 12, 3327–3328, 2040007. 2040007

[5] Kees U. R. , Mulcahy M. T. , and Willoughby M. L. , Intrathoracic Carcinoma in an 11-Year-Old Girl Showing a Translocation t (15; 19), American Journal of Pediatric Hematology/Oncology. (1991) 13, no. 4, 459–464, 10.1097/00043426-199124000-00011. 1785673

[6] French C. A. , Ramirez C. L. , Kolmakova J. , Hickman T. T. , Cameron M. J. , Thyne M. E. , Kutok J. L. , Toretsky J. A. , Tadavarthy A. K. , Kees U. R. , Fletcher J. A. , and Aster J. C. , BRD-NUT Oncoproteins: A Family of Closely Related Nuclear Proteins That Block Epithelial Differentiation and Maintain the Growth of Carcinoma Cells, Oncogene. (2008) 27, no. 15, 2237–2242, 10.1038/sj.onc.1210852, 17934517. 17934517

[7] Suzuki S. , Kurabe N. , Ohnishi I. , Yasuda K. , Aoshima Y. , Naito M. , Tanioka F. , and Sugimura H. , NSD3-NUT-Expressing Midline Carcinoma of the Lung: First Characterization of Primary Cancer Tissue, Pathology-Research and Practice. (2015) 211, no. 5, 404–408, 10.1016/j.prp.2014.10.013, 25466466. 25466466

[8] Chen M. , Zhao S. , Liang Z. , Wang W. , Zhou P. , and Jiang L. , NUT Carcinoma of the Parotid Gland: Report of Two Cases, One With a Rare ZNF532-NUTM1 Fusion, Virchows Archiv. (2022) 480, no. 4, 887–897, 10.1007/s00428-021-03253-9, 35064291. 35064291

[9] Shiota H. , Elya J. E. , Alekseyenko A. A. , Chou P. M. , Gorman S. A. , Barbash O. , Becht K. , Danga K. , Kuroda M. I. , Nardi V. , and French C. A. , ‘Z4’ Complex Member Fusions in NUT Carcinoma: Implications for a Novel Oncogenic Mechanism, Molecular Cancer Research. (2018) 16, no. 12, 1826–1833, 10.1158/1541-7786.MCR-18-0474, 30139738. PMC6279489 30139738

[10] Kloker L. D. , Sidiras M. , Flaadt T. , Brecht I. B. , Deinzer C. K. W. , Groß T. , Benzler K. , Zender L. , and Lauer U. M. , Clinical Management of NUT Carcinoma (NC) in Germany: Analysis of Survival, Therapy Response, Tumor Markers and Tumor Genome Sequencing in 35 Adult Patients, Lung Cancer. (2024) 189, 107496, 10.1016/j.lungcan.2024.107496, 38301600. 38301600

[11] Stevens T. M. , Morlote D. , Xiu J. , Swensen J. , Brandwein-Weber M. , Miettinen M. M. , Gatalica Z. , and Bridge J. A. , NUTM1-Rearranged Neoplasia: A Multi-Institution Experience Yields Novel Fusion Partners and Expands the Histologic Spectrum, Modern Pathology. (2019) 32, no. 6, 764–773, 10.1038/s41379-019-0206-z, 30723300. PMC8194366 30723300

[12] Kotekar A. , Singh A. K. , and Devaiah B. N. , BRD4 and MYC: Power Couple in Transcription and Disease, FEBS Journal. (2023) 290, no. 20, 4820–4842, 10.1111/febs.16580, 35866356. PMC9867786 35866356

[13] Giridhar P. , Mallick S. , Kashyap L. , and Rath G. K. , Patterns of Care and Impact of Prognostic Factors in the Outcome of NUT Midline Carcinoma: A Systematic Review and Individual Patient Data Analysis of 119 Cases, European Archives of Oto-Rhino-Laryngology. (2018) 275, no. 3, 815–821, 10.1007/s00405-018-4882-y, 29356890. 29356890

[14] Cho Y. A. , Choi Y. , Hwang I. , Lee K. , Cho J. H. , and Han J. , Clinicopathological Characteristics of Primary Lung Nuclear Protein in Testis Carcinoma: A Single-Institute Experience of 10 Cases, Thoracic Cancer. (2020) 11, no. 11, 3205–3212, 10.1111/1759-7714.13648, 33009876. PMC7606005 33009876

[15] Farooq A. , Kerper A. L. , Boland J. M. , and Lo Y. C. , Nuclear Protein in Testis (NUT) Carcinoma: A Comprehensive Immunohistochemical Analysis of 57 Cases With Consideration of Interpretation and Pitfall Recognition, Archives of Pathology & Laboratory Medicine. (2024) 148, no. 8, 898–904, 10.5858/arpa.2023-0178-OA, 38059551. 38059551

[16] Sahai V. , Redig A. J. , Collier K. A. , Eckerdt F. D. , and Munshi H. G. , Targeting Bet Bromodomain Proteins in Solid Tumors, Oncotarget. (2016) 7, no. 33, 53997–54009, 10.18632/oncotarget.9804, 27283767. PMC5288238 27283767

[17] Ye Z. , Li X. , Zhang M. , Xie F. , Luo X. , Deng C. , Yang D. , and Yin M. , Complete Response to BET Inhibitor in Primary Pulmonary NUT Carcinoma With Single-Cell Sequencing-Based Analysis: A Case Report, JTO Clinical and Research Reports. (2025) 6, no. 10, 100885, 10.1016/j.jtocrr.2025.100885. PMC12414819 40926997

[18] Kaplan H. G. , Subramaniam S. , Vallières E. , and Barnett T. , Prolonged Survival of NUT Midline Carcinoma and Current Approaches to Treatment, Oncologist. (2023) 28, no. 9, 765–770, 10.1093/oncolo/oyad177, 37311046. PMC10485280 37311046

[19] Napolitano M. , Venturelli M. , Molinaro E. , and Toss A. , NUT Midline Carcinoma of the Head and Neck: Current Perspectives, OncoTargets and Therapy. (2019) 12, 3235–3244, 10.2147/OTT.S173056, 31118674. PMC6501778 31118674

