# Supplementary material for: Two Cases of NUT Carcinoma of the Lung: Same Name With Different Appearance: A Case Report
Source: Case Rep Pathol. 2026 Oct 6;2026:9648746. doi: 10.1155/crip/9648746 (PMC13640534; doi:10.1155/crip/9648746)
Supplement: Supplementary file 1 [file CRIP-2026-9648746-s001.pdf]

CARE case report guidelines

CARE Checklist of information to include when writing a case report

(cc) BY-NC-ND

Topic	Item	Checklist item description	Reported on Line
Title	1	The diagnosis or intervention of primary focus followed by the words "case report"	1-2
Key Words	2	2 to 5 key words that identify diagnoses or interventions in this case report, including "case report"	52-53
Abstract (no references)	3a	Introduction: What is unique about this case and what does it add to the scientific literature?	40-42, 47-49
	3b	Main symptoms and/or important clinical findings	31-40
	3c	The main diagnoses, therapeutic interventions, and outcomes	40-42
	3d	Conclusion—What is the main "take-away" lesson(s) from this case?	42-47
Introduction	4	One or two paragraphs summarizing why this case is unique (**may include references**)	61-63
Patient Information	5a	De-identified patient specific information.	69-70, 103
	5b	Primary concerns and symptoms of the patient.	69-70, 103
	5c	Medical, family, and psycho-social history including relevant genetic information	69-70
	5d	Relevant past interventions with outcomes	none
Clinical Findings	6	Describe significant physical examination (PE) and important clinical findings.	70-73, 103-104
Timeline	7	Historical and current information from this episode of care organized as a timeline	90-91, 126-132
Diagnostic Assessment	8a	Diagnostic testing (such as PE, laboratory testing, imaging, surveys).	70-72, 103-104
	8b	Diagnostic challenges (such as access to testing, financial, or cultural)	not obvious
	8c	Diagnosis (including other diagnoses considered)	76-100, 104-125
	8d	Prognosis (such as staging in oncology) where applicable	90-91, 131-132
Therapeutic Intervention	9a	Types of therapeutic intervention (such as pharmacologic, surgical, preventive, self-care)	90, 109-110, 126-132
	9b	Administration of therapeutic intervention (such as dosage, strength, duration)	128-130
	9c	Changes in therapeutic intervention (with rationale)	130-131
Follow-up and Outcomes	10a	Clinician and patient-assessed outcomes (if available)	
	10b	Important follow-up diagnostic and other test results	95-100, 122-125
	10c	Intervention adherence and tolerability (How was this assessed?)	130-131
	10d	Adverse and unanticipated events	
Discussion	11a	A scientific discussion of the strengths AND limitations associated with this case report	199-204
	11b	Discussion of the relevant medical literature **with references**.	135-209
	11c	The scientific rationale for any conclusions (including assessment of possible causes)	164-192
	11d	The primary "take-away" lessons of this case report (without references) in a one paragraph conclusion	203-204, 208-209
Patient Perspective	12	The patient should share their perspective in one to two paragraphs on the treatment(s) they received	not available
Informed Consent	13	Did the patient give informed consent? Please provide if requested	Yes ☑ No ☐

We obtained informed consent from the patients' next of kin, as both patients were deceased.
